# Spontaneous Coronary Artery Dissection of an Anomalous Right Coronary Artery in a Young Male

**DOI:** 10.7759/cureus.16924

**Published:** 2021-08-05

**Authors:** Noor Ul Ann Rabbani, Kanaan Mansoor, Mohammed I Ranavaya, Jason Mader, Melissa D Lester

**Affiliations:** 1 Internal Medicine, Marshall University Joan C. Edwards School of Medicine, Huntington, USA; 2 Cardiology, Marshall University Joan C. Edwards School of Medicine, Huntington, USA; 3 General Surgery, University of Louisville, Louisville, USA

**Keywords:** spontaneous coronary dissection, anomalous rca, non st-elevation acute coronary syndrome, young adult male, coronary artery bypass grafting (cabg)

## Abstract

Spontaneous coronary artery dissection (SCAD) is a tear in the coronary artery layers that presents clinically as an acute coronary syndrome (ACS), ventricular arrhythmias, or sudden cardiac death (SCD). It is uncommon for young healthy males with no comorbid conditions to have SCAD. We report an interesting case of SCAD in an anomalous right coronary artery (RCA) in a young 33-year-old male. The patient presented with episodes of midsternal chest pain and had elevated troponins on laboratory workup. A left heart catheterization revealed anomalous RCA, originating from the left aortic sinus. The left heart catheterization also demonstrated a SCAD of the anomalous RCA. Cardiothoracic surgery was consulted, and the patient had placement of saphenous vein graft to the proximal RCA. While this patient’s presentation of ACS in the setting of SCAD is relatively common, it was atypical due to gender and lack of precipitating stressors. One of the risk factors this patient did have was the anomalous RCA arising from the left aortic sinus. There is scarce literature involving guidance for therapeutic intervention for RCA ostial lesion, let alone an anomalous one. Although coronary artery bypass grafting (CABG) remains the most clinically sound decision, in this case, further development of guidelines for RCA lesions would aid in decision-making.

## Introduction

Spontaneous coronary artery dissection (SCAD) is the acute separation of the coronary artery layers that are not associated with trauma, atherosclerosis, or iatrogenic damage. It is believed to be secondary to spontaneous medial separation or vasa vasorum rupture which results in an intramural hematoma [1]. It most commonly occurs in middle-aged women and is also associated with postpartum myocardial infarction [1]. Other risk factors include emotional stress, physical stress, and illicit drug use, including stimulants [2]. Herein, we describe a 33-year-old male patient who presented with non-ST segment elevation myocardial infarction (NSTEMI) due to SCAD close to the ostium of an anomalous right coronary artery (RCA) originating off the left aortic sinus.

## Case presentation

**Figure 1:**
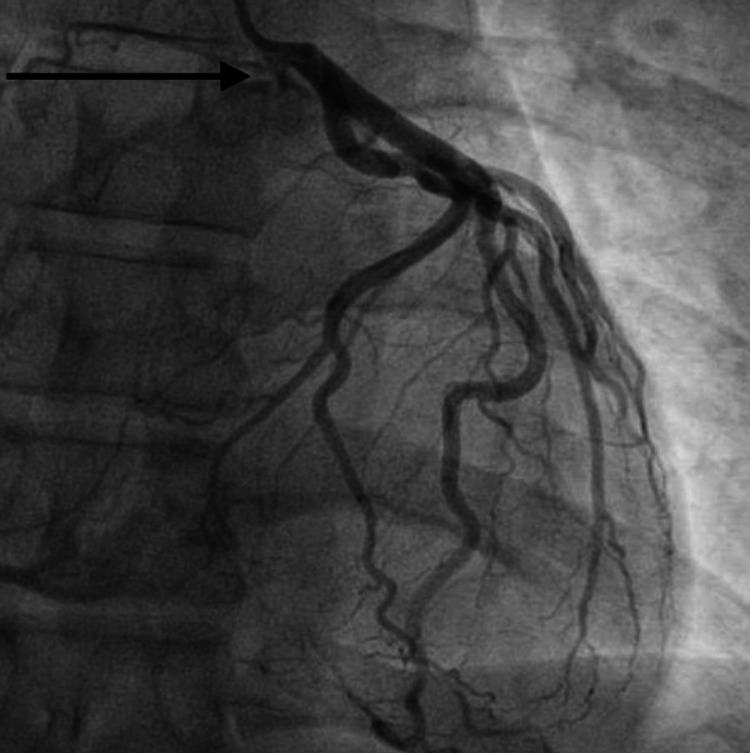
Anomalous right coronary artery originated from the left aortic sinus

**Figure 2:**
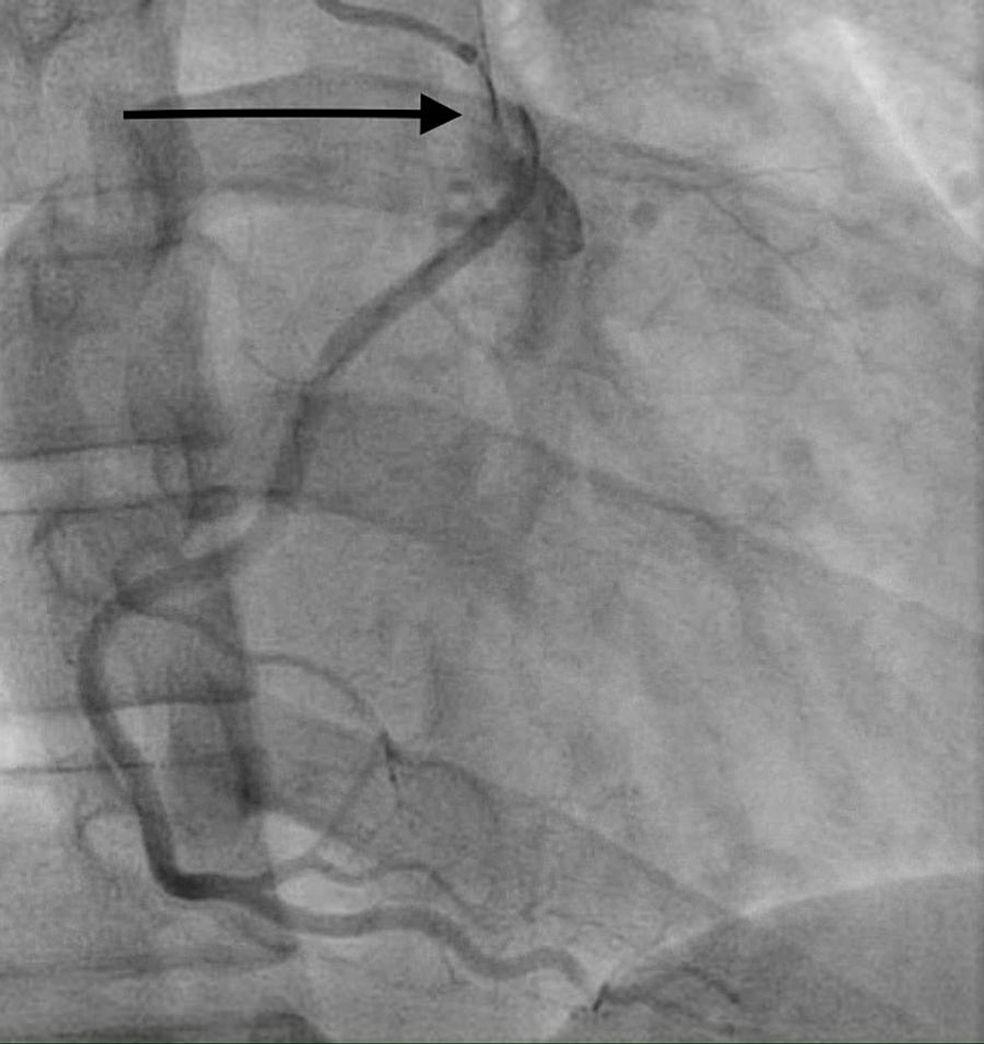
Right coronary artery ostial dissection

**Figure 3:**
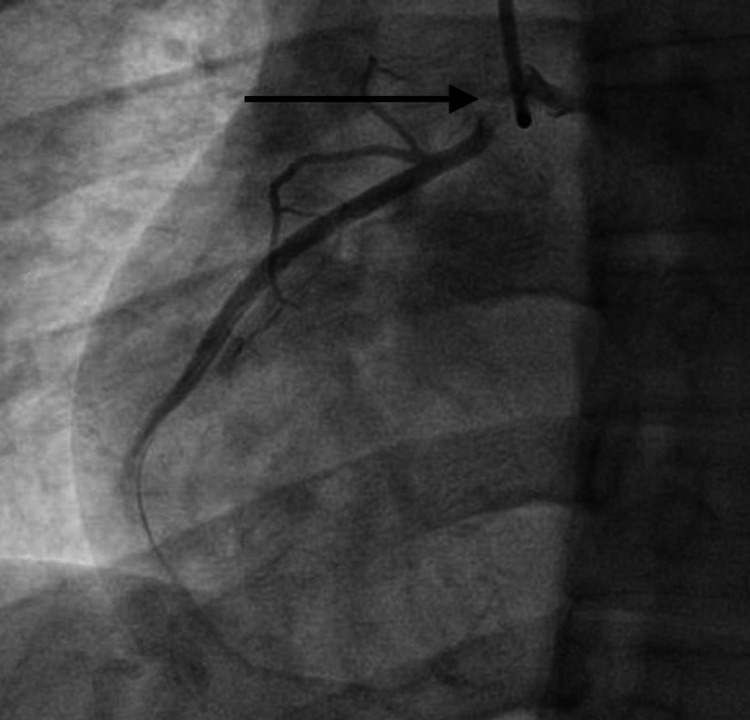
Right coronary artery ostial dissection

A 33-year-old male with no known comorbid conditions presented with intermittent chest pain. He described the chest pain as a pressure-like sensation in the midsternal area. He denied radiation of the chest pain to other areas of the chest wall, left arm, or jaw. The patient did not report any worsening of chest pain with cough, taking a deep breath, or movement. The patient had two episodes of chest pain in the past two days and each episode lasted 10-15 minutes. The patient denied any similar episodes of chest pain in the past or any familial cardiac history. The patient denied any history of smoking or alcohol abuse. He denied using illicit drugs including intravenous drugs. He denied ever having an echocardiogram or heart catheterization. Vital signs were stable and physical examination revealed no chest wall tenderness. Electrocardiogram (ECG) showed sinus rhythm with no significant ST changes. The patient had a basic laboratory workup done that included complete blood count (CBC), comprehensive metabolic panel (CMP), and troponin level. High sensitivity troponin level was significantly elevated to 704.6 pg/mL (normal range: 3.0-58.0 pg/mL). The remainder of his basic laboratory workup was within normal limits. He was started on a weight-based heparin drip and taken to the cardiac catheterization lab. The left heart catheterization report revealed normal anatomy of the left coronary artery (LCA), originating from the left aortic sinus, bifurcating into the left anterior descending (LAD) artery, and left circumflex artery (LCX). The RCA was originating from an anomalous position, adjacent to the LCA, but slightly anterior to it. The RCA originated from the left aortic sinus (Figure 1). There was an ostial dissection of the anomalous RCA (Figures 2, 3). Cardiothoracic surgery was consulted and recommended coronary artery bypass grafting (CABG). The patient underwent CABG with the placement of a saphenous vein graft to the proximal RCA. The patient’s postoperative hospital course was uneventful with no complications. He had complete resolution of his symptoms and was discharged in stable condition.

## Discussion

SCAD is a life-threatening condition that presents as an acute coronary syndrome (ACS), ventricular arrhythmias, or sudden cardiac death (SCD) [1]. It is most often seen in females, up to 91% of the time [3]. Previously, it was thought to be mainly associated with pregnancy and the puerperal period. However, this does not appear to be the case, as most women develop this condition during the age range of 47-53 [1]. Regardless of gender, risk factors are often present and provide clues to the underlying etiology of this disorder. It has been demonstrated that SCAD may be the initial presentation of fibromuscular dysplasia [1]. Additionally, illnesses, such as autoimmune or connective tissue disorders, such as Marfan’s syndrome, potentially weaken the walls of arteries and allow either physical or emotional stressors to precipitate the intimal tear leading to SCAD [3]. Further, drugs, such as methamphetamine and cocaine, have been implicated and general use of recreational drugs has been loosely associated with SCAD [2].

While this patient's presentation of NSTEMI in the setting of SCAD is relatively common, it was atypical due to the gender and lack of precipitating stressors. He lacked typical risk factors, denied smoking, was not using vasoactive drugs, and did not demonstrate the physical stigmata of connective tissue disorders or autoimmune diseases. He also did not have illnesses commonly found in those with SCAD including diabetes mellitus, dyslipidemia, and hypertension [4]. Importantly, this shows that despite the female predominance of SCAD and its relatively uncommon nature, consider SCAD in the differential for any young patient presenting with an ACS syndrome.

It should be noted that one of the risk factors this patient did possess was the anomalous RCA arising from the left anterior sinus. Although rare in itself, this is more common than the LCA arising from the right sinus [5]. While there is clear clinical significance and risk for SCD via an LCA arising from the right sinus, there is ambiguity as to the clinical risk that the RCA arising from the left aortic sinus presents. This pathology has been found in cases of SCD on necropsy in several studies; however, one study of Japanese patients, 44 of whom had the pathology, demonstrated no deaths from SCD and symptom control with medical management only [6,7].

The diagnosis of SCAD revolves around identifying characteristics of dissection either on conventional or CT coronary angiography [1,8]. Intravascular ultrasound (IVUS) can also be used to clarify the type of SCAD; however, it was avoided in this case due to fear of propagating the dissection, and clear coronary angiogram findings. While the diagnosis is straightforward, the treatment options remain ambiguous [8]. Because this presents as an ACS, initial treatment involves immediate therapy for myocardial infarction. Once SCAD is diagnosed or suspected, further management depends upon both the dissection type and risk factors. Medical management is a viable option when the patient is hemodynamically stable, their status suggests stability, and the dissection does not involve multiple vessels, the LAD ostium, and LCA [1,4]. When the patient does not meet the criteria for medical management, then percutaneous coronary intervention (PCI) or CABG are alternative options [3]. While the literature suggests a LAD ostial lesion is a high risk, there is scarce literature involving guidance for therapy in an RCA ostial lesion, let alone an anomalous one. Based on the patient’s symptoms and clinical status, our patient would have been a candidate for medical management; however, the location of the dissection at the ostium of the anomalous RCA was concerning. Its location made a PCI approach difficult, thus a decision was made to proceed with CABG with the placement of venous graft bypassing the dissection.

## Conclusions

SCAD is a rare entity that should be considered in the differential for ACS, especially in the case of young individuals. In this case, the patient did not have any typical risk factors and SCAD was diagnosed upon performing cardiac catheterization. Although CABG remains the most clinically sound decision in this case due to the anomalous RCA ostial involvement, further development of guidelines for RCA lesions would aid in the decision-making.
